# Computer-Aided Diagnosis of Alzheimer’s Disease through Weak Supervision Deep Learning Framework with Attention Mechanism

**DOI:** 10.3390/s21010220

**Published:** 2020-12-31

**Authors:** Shuang Liang, Yu Gu

**Affiliations:** 1 School of Automation and Electrical Engineering, University of Science and Technology Beijing, Beijing 100083, China; liangshuang@xs.ustb.edu.cn; 2 School of AutoMation, Guangdong University of Petrochemical Technology, Maoming 525000, China; 3 Beijing Advanced Innovation Center for Soft Matter Science and Engineering, Beijing University of Chemical Technology, Beijing 100029, China; 4 Department of Chemistry, Institute of Inorganic and Analytical Chemistry, Goethe-University, 60438 Frankfurt, Germany

**Keywords:** Alzheimer’s disease, attention module, CNN, computer-aided diagnosis, magnetic resonance imaging, multi-task learning, weakly supervised learning

## Abstract

Alzheimer’s disease (AD) is the most prevalent neurodegenerative disease causing dementia and poses significant health risks to middle-aged and elderly people. Brain magnetic resonance imaging (MRI) is the most widely used diagnostic method for AD. However, it is challenging to collect sufficient brain imaging data with high-quality annotations. Weakly supervised learning (WSL) is a machine learning technique aimed at learning effective feature representation from limited or low-quality annotations. In this paper, we propose a WSL-based deep learning (DL) framework (ADGNET) consisting of a backbone network with an attention mechanism and a task network for simultaneous image classification and image reconstruction to identify and classify AD using limited annotations. The ADGNET achieves excellent performance based on six evaluation metrics (Kappa, sensitivity, specificity, precision, accuracy, F1-score) on two brain MRI datasets (2D MRI and 3D MRI data) using fine-tuning with only 20% of the labels from both datasets. The ADGNET has an F1-score of 99.61% and sensitivity is 99.69%, outperforming two state-of-the-art models (ResNext WSL and SimCLR). The proposed method represents a potential WSL-based computer-aided diagnosis method for AD in clinical practice.

## 1. Introduction

Alzheimer’s disease (AD) is a common chronic progressive neurodegenerative disease of the elderly characterized by progressive dementia and brain degeneration. It significantly affects cognitive functions, memory functions, the quality of life, and the emotions of more than 50 million people worldwide [1]. According to a report by the World Health Organization (WHO), AD has become the fifth leading cause of death, and the number of AD patients will increase to 152 million by 2050, and by 2050 [2]. However, the etiology of AD remains unclear, and there are no effective drugs or treatments to reverse dementia [3]. The preclinical stage of AD, called mild cognitive impairment (MCI), is a transitional state between normal aging and AD [4]. According to a report of the American Academy of Neurology [5], about 10% to 15% of patients with MCI may eventually suffer from AD, whereas only 1% to 2% of patients experience normal aging. Unfortunately, due to a lack of understanding of AD by patients and their family members, most patients suffer from moderate and severe stages of AD at the time of diagnosis and have missed the optimal intervention stage [6]. Therefore, it is of great significance to identify the risk and extent of AD as early as possible. Typically, doctors have to conduct careful medical assessments of patients, such as neuropsychological examinations and neuroimaging, to identify the risk and extent of developing dementia [7].

As a result of significant progress in neuroimaging technology, characteristic changes can now be observed in the brains of patients with AD, including changes in the prodromal and presymptomatic states, providing information for doctors to obtain a more accurate diagnosis [8]. Different forms of neuroimaging techniques have been used in clinical practice to diagnose AD, including computed tomography (CT), positron emission tomography (PET), and magnetic resonance imaging (MRI) [9]. CT is a structural imaging technique that integrates X-ray projections from multiple angles and generates cross-sectional or three-dimensional (3D) images [10]. It has the advantages of low cost and fast examination speed. However, the resolution of the medial temporal lobe is relatively low, which may lead to MCI being misdiagnosed as a normal aging symptom [11]. PET is another structural imaging technique that provides useful information for the diagnosis of AD by detecting the distribution of positron nuclide markers for metabolic information [12]. However, both CT and PET examinations expose the patients to radiation, whereas MRI has the unique advantage of not causing radiation damage [13]. MRI is a medical imaging technique that uses electromagnetic signals obtained from the human body by magnetic resonance to generate images of organs [14]. Moreover, it is highly sensitive to brain contraction and can be used to construct 3D brain tissue images at high resolution [15]. Therefore, it is promising to use MRI to understand and diagnose AD in clinical practice. With the rapid development and wide application of artificial intelligence (AI) in the medical field, computer-aided diagnosis (CAD) of AD using neuroimaging may be an auxiliary method to assist physicians. Here, CAD can be regarded as an image understanding and classification problem. Deep learning (DL), in particular convolutional neural networks (CNNs), has proved to be an effective method of feature extraction from images and has provided state-of-the-art (SOTA) solutions in different image understanding and recognition tasks. Various DL-based methods for CAD have also been developed. Mansour et al. used AlexNet [16] for diagnosing diabetic retinopathy and achieved an accuracy of 97.93% [17]. Yang et al. designed a patch-based DL framework to detect prostate cancer using MRI data and achieved a specificity of 90.6% [18]. Zhu et al. proposed a landmark-based feature representation method and employed a CNN model for the diagnosis of AD with an accuracy of 91.57% [19]. These methods are supervised learning methods that require a large sample size and high-quality manually annotated data for accurate feature representation [20]. However, it is time-consuming and costly to obtain medical images along with high-quality annotations in practical applications. Therefore, the development of a weakly supervised learning (WSL) method is of great significance to mine massive amounts of medical image data at a low cost and with high accuracy. Mahajan et al. presented a WSL method using ResNext [21] as the backbone network and trained the model using images from the Instagram website for pretraining. The hashtags were used as labels, and the pre-trained model was fine-tuned on the ImageNet dataset. The model achieved a top-1 accuracy of 85.4% on the ImageNet-1k benchmark [22]. In 2020, Hinton et al. presented the simple framework SimCLR for contrastive learning of visual representations and trained the SimCLR in a self-supervised learning manner. The SimCLR achieved a high accuracy of 85.8%, using only 1% of the labels of the ImageNet [23].

This paper proposes a WSL-based DL framework for the identification and classification of AD. The proposed framework consists of two parts, i.e., the backbone network with an attention mechanism and the task networks. This paper provides the following contributions:

1. An attention module (AM) is proposed to improve the discriminative ability of the backbone network with a low computational cost. The AM is an automatic weighting module that adjusts the weights of the channels in the feature maps so that the backbone network selectively focuses on the significant parts of the input.
2. The task networks perform two tasks (image classification and image reconstruction) in parallel. The task networks utilize the feature vector generated by the backbone network and use fully-connected (FC) layers and a decoder for label prediction and image reconstruction.
3. A multi-task learning (MTL) framework is proposed for conducting image recognition and reconstruction in parallel, with low computational requirements and good performance (with the best F1-score of 99.61% and a sensitivity of 99.69%) using only 20% of the labels from the datasets for fine-tuning.

The rest of the paper consists of five parts. The related studies are described in Section 2. The proposed method is explained in Section 3. Section 4 summarizes the results. The discussion is presented in Section 5, and the conclusion is given in Section 6.

## 2. Related Works

### 2.1. Multi-Task Learning

Multi-task learning is a transfer learning method that extracts domain-specific information from related tasks for an improved representation of the input data [24]. The concept of MTL was first proposed by Caruana et al. [25] and has been applied in many fields. Various studies have been conducted to explore effective MTL methods. Misra et al. built a novel sharing unit to learn representation from different tasks and reported excellent results [26]. Lu et al. developed an adaptive feature sharing mechanism in MTL to identify different attributes of people [27]. In the above studies, each task has a same priority. While some studies focused on a single task, whereas others acted as auxiliary tasks. Auxiliary tasks, which provide additional information, provide valid information from aspects of the main task. Zhang et al. used head pose estimation and attribute prediction of faces as auxiliary tasks and facial landmark detection as the main task; it was found that the precision of this method was higher than that of other methods [28]. Our framework belongs to this type of transfer learning method.

### 2.2. Weakly Supervised Learning

It is well-known that supervised learning-based models require a large amount of well-labeled data to obtain accurate predictions. In contrast, unsupervised learning-based models typically lack high precision, and the learning process is less effective than that of supervised learning methods [29]. Weakly supervised learning is a machine learning technique with the objective of learning effective feature representation from limited or low-quality annotations [30]. Various WSL-based methods have been explored in different fields. Hu et al. proposed a CNN-based WSL framework for the task of multimodal image registration and achieved STOA performance [31]. Wang et al. developed a WSL-based method for accurate automated segmentation of remote sensing data with a proposed U-Nets framework and obtained superior segmentation performance [32]. ResNeXt WSL is the most recent WSL method. It was used to pre-train images from the Instagram website, followed by fine-tuning on the ImageMet dataset [22]. SimCLR, an unsupervised learning method, was used in combination with WSL and achieved SOTA performance [23].

### 2.3. Image Classification

The objective of image classification is to classify an image or instance into categories [33]. With the rapid development and verification of CNNs, various CNN-based frameworks have been developed and used for image classification [34]. LeCun et al. first develop the CNN framework (LeNet) for document recognition [35]. AlexNet, which is an improvement of LeNet and surpassed traditional machine learning methods, won the Imagenet competition in 2012 [16]. Since then, different types of CNN architectures have been proposed, such as ResNet [36], ResNext [21], and InceptionNet [37]. In this study, we adopted the structure of the residual block, which is used in ResNet [36].

### 2.4. Image Reconstruction

Image reconstruction, which is a critical problem in medical imaging, is a technique for creating 2D or 3D images from sets of 1D projections [38]. The autoencoder is the most popular technique and has proved effective in image reconstruction of unlabeled images [39]. In this study, we designed a sub-network to perform image reconstruction using abundant features.

## 3. Materials and Methods

### 3.1. The Pipeline of the Proposed Framework

**Figure 1:**
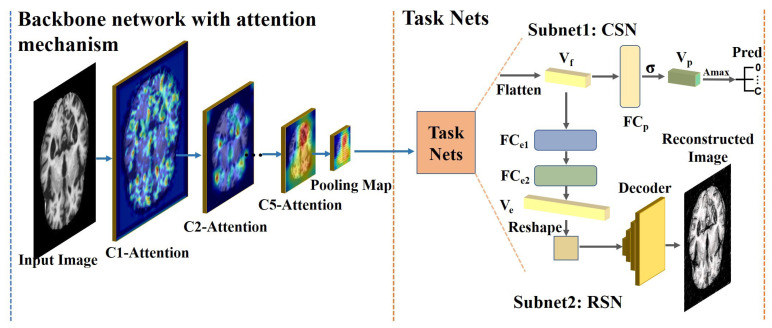
The proposed framework. The framework consists of two parts, including a backbone network with an attention mechanism that acts as a shared network for extracting salient features and task nets that contain two sub-networks. The two sub-networks simultaneously conduct two sub-tasks, i.e., classification and reconstruction.

The proposed WSL-based DL framework, which is called ADGNET, is a CNN-based single-input-multi-output (SIMO) architecture consisting of two components: an improved backbone network with the attention mechanism and task nets that consists of two sub-networks, i.e., the classification sub-network (CSN) and the reconstruction sub-network (RSN). The backbone network has a residual network structure with the proposed AM to obtain highly discriminative representations while suppressing unrelated regions in the images. As shown in Figure 1, the backbone network consists of five convolution stages (C1-Attention to C5-Attention), followed by the Resnet. Generally, feature maps generated by the deeper stages contain more semantic information, and those generated by the shallower stages contain more detailed information, such as edges and corners. The backbone network extracts features step-by-step from the MRI input images and generates a pooling map using global average pooling (GAP). The task nets use the pooling map as input and flatten it as a feature vector $V_{f}$. The $V_{f}$ is then sent to two different task branches; one generates the prediction vector $V_{p}$ using the *FC* layer, and the other reconstructs the original images using the *FC* layers and a decoding module. As shown in Figure 1, the $V_{p}$ is sent to the *argmax* (Amax), which returns the index with the largest value of the axes of the $V_{p}$ and provides the classification results. Here *C* denotes the number of categories.

### 3.2. Backbone Network with the Proposed Attention Module

**Figure 2:**
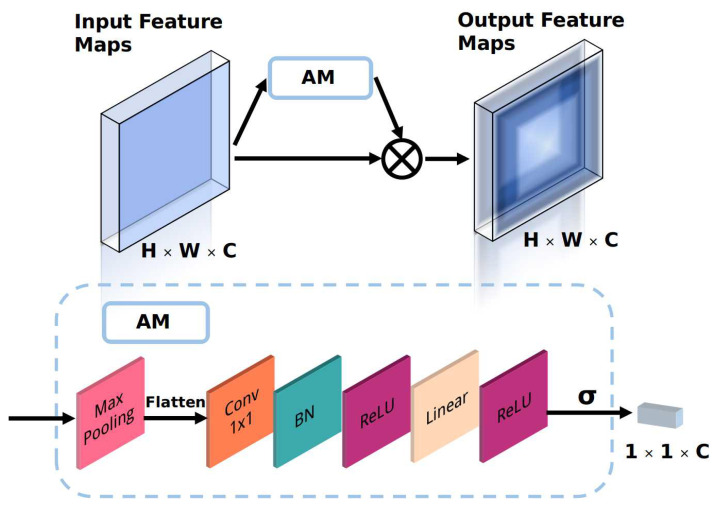
The proposed attention module.

The backbone network is a multi-stage convolution network that follows the Resnet to avoid the gradient vanishing problem. Different stages in the backbone network generate feature maps with different resolutions. Notably, the backbone network is a convolution network shared by the two sub-task nets, providing a parameter-efficient and time-efficient method. In the reconstruction task, the backbone network can be regarded as part of an encoder network that automatically learns the feature representation from the images without annotation information. In the classification task, the backbone network can be considered a feature extractor, which is optimized using the supervised learning principle. As described in Section 3.1, each stage of the backbone network generates the attention feature maps after implementing the proposed *AM*. As shown in Figure 2, the input feature maps $F_{i}^{s}$ with a size of H×W×C and a scale of *s* at layer *i* are the output of the ith stage of Resnet. Given the $F_{i}^{s}$ as input, the *AM* outputs the channel attention factors (*CAF*) with a size of 1×1×C so that the network can automatically determine the importance of the extracted features.This process can also be regarded as a feature filtering and selection method that improves the discrimination ability of the network at low computational cost. The *CAF* are then fused with the $F_{i}^{s}$ using the element-wise multiplication operation (EWMO), and the fused feature map $F_{i}^{s^{\prime }}$ is the output. The feature extraction process of the backbone network can be expressed as follows:

(1) $$\begin{array}{cc} CAF & =AM(F_{i}^{s})\\ F_{i}^{s^{\prime }} & =CAF⊗F_{i}^{s} \end{array}$$

where *AM* is the proposed attention module, and ⊗ is the EWMO.

The *AM* is an automatic weighting module that learns the channel weights of the input feature maps. As shown in Figure 2, the input $F_{i}^{s}$ is first downsampled using the global max-pooling operation to retain important information while reducing the computational cost. The downsampled feature maps are then flattened into a one-dimensional vector. The flattened vector is then sent to a convolution layer with a 1×1 kernel to extract features from the vector and adjust its dimension. The extracted features are sent to the batch norm (BN) layer and activated using the ReLU function to speed up the training and convergence speed of the module and increase its nonlinear representation ability. After these operations, an *FC* layer (Linear) with ReLU as the activation function is adopted to output the *CAF* with a size of 1×1×C. The final output is generated using the sigmoid function to convert the values of the *CAF* to a range of 0 to 1; these values can be considered the importance scores of the channels in the $F_{i}^{s}$.

### 3.3. Task Sub-Networks

**Figure 3:**
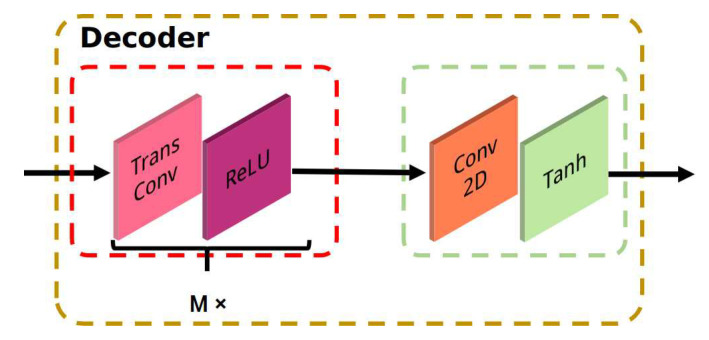
Details of the proposed decoder component. The decoder consists of two parts, including a transposed convolution layer with a ReLU activation function and a convolution with a convolution layer and a Tanh layer.

As shown in Figure 1, the task sub-networks consist of the CSN and the RSN. The input to the two parts is the flattened vector ($V_{f}$). The two tasks are performed in parallel. The CSN is a simple *FC* layer called $FC_{p}$ that generates a vector with a size of 1×C. The vector is then sent to the sigmoid function to generate the prediction vector ($V_{p}$) with a range of 0 to 1 that is used as a probability of prediction of the specified classes. The process of the CSN can be formulated as follows:

(2) $$\begin{array}{cc} V_{p} & =\sigma (FC\left(V_{f}\right)) \end{array}$$

where *FC* is the *FC* layer $FC_{p}$; σ is the sigmoid function. The RSN consists of two components, i.e., the encoder and the decoder. The encoder is constructed using the backbone network and two *FC* layers called $FC_{e1}$ and $FC_{e2}$. The backbone network extracts and abstracts the features step-by-step, and the two *FC* layers encode the features into a vector ($V_{e}$). The decoder component is a multi-layer transposed convolution network. The details of the decoder component are shown in Figure 3. The input of the decoder component is the $V_{e}$ after the reshape operation, which converts the $V_{e}$ to a two-dimensional feature map. The decoder is a modular network consisting of two parts with multiple transposed convolution layers. Each transposed convolution layer in the decoder has multiple transposed convolution kernels with a size of 3×3, a stride of 2, and a padding of 1. As shown in Figure 3, there are M× transposed convolution layers and ReLU layers in the first part, which decode the input feature maps and up-sample the input. The second part of the encoder contains a convolution layer and a Tanh layer. The convolution layer is used for dimension normalization to convert the feature maps to the same size as the input MRI image. The Tanh layer is used to output the predicted MRI image since it has a wide range of predicted values, improving the prediction accuracy. The RSN process can be formulated as follows:

(3) $$\begin{array}{cc} V_{e} & =FC_{e2}\left(FC_{e1}\left(V_{f}\right)\right)\\ Im_{r} & =\tanh(Dec\left(V_{e}\right)) \end{array}$$

where $FC_{e1}$ and $FC_{e2}$ are the *FC* layers; tanh is the tanh function. *Dec* is the proposed decoder part and $Im_{r}$ is the reconstructed image.

### 3.4. Loss Function

The proposed ADGNET can be trained in an end-to-end manner. The loss function of the framework is composed of two parts and is defined as follows:

(4) $$\begin{array}{cc} L & =\lambda_{1}L_{cls}+\lambda_{2}L_{rec} \end{array}$$

where $L_{cls}$ and $L_{rec}$ are the classification loss and the image reconstruction loss, respectively. $\lambda_{1}$ and the $\lambda_{2}$ are the weighting factors which balance the two losses.

As we described in the introduction, it is difficult to distinguish dementia in the early and middle stages from normal aging because of the small differences in brain imaging. Thus, we adopted the *focus* idea, as introduced in previous works [40,41], to ensure that the framework focuses primarily on difficult and misclassified samples. We proposed a new loss function based on the cross-entropy loss. The modified classification loss is defined as follows:

(5) $$\begin{array}{c} L_{cls}=-\frac{1}{N}\left({\left(1-i\right)}^{\gamma_{i}}log\left(1-i^{{}^{\prime }}\right)+i^{\gamma_{i}}log\left(i^{{}^{\prime }}\right)\right) \end{array}$$

where *N* is the number of samples participating in a single optimization. $\gamma_{i}$ represents the class-wise weight reduction factors, which adjust the importance of different samples for an improved representation. *i* is the ground truth probability of a target belonging to a given class, and $i^{{}^{\prime }}$ is the prediction probability of the target belonging to the given class.

The new loss function is a modification based on the cross-entropy (CE) loss. As shown in Figure 1, the final probability of a sample be a specified category is generated using the sigmoid function which range from 0 to 1. Therefore, the equation 5 demonstrated the loss for each specified category following the formulation of the CE loss, and the class-wise weight reduction factors $\gamma_{i}$ can be considered as a numerical vector. In our manuscript, the values of $\gamma_{i}$ were all set as 2 as default.

We used the mean square error function as the reconstruction loss; it is defined as follows:

(6) $$\begin{array}{c} L_{rec}=-\frac{1}{N}\sum_{i=1}^{N}{\left(Y_{i}-\hat{Y_{i}}\right)}^{2} \end{array}$$

where *N* is the number of samples participating in a single optimization. $Y_{i}$ is the ground truth value of the input sample, and $\hat{Y_{i}}$ is the prediction value of the reconstructed sample.

## 4. Results

### 4.1. Multi-Modal Brain Imaging Dataset

**Figure 4:**
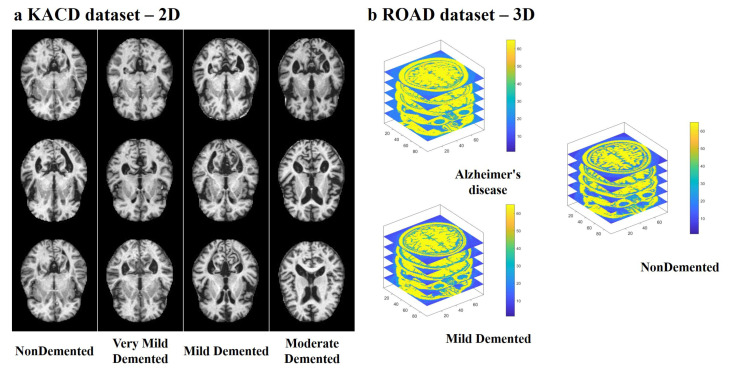
Examples of multi-modal data; (**a**) magnetic resonance imaging (MRI) images (2D) from the KACD dataset, (**b**) MRI images (3D) from the ROAD dataset.

In this study, the proposed ADGNET was evaluated on two different brain imaging datasets for a comprehensive assessment. The two brain imaging datasets are the Kaggle Alzheimer’s classification dataset (KACD) [42] and the Recognition of Alzheimer’s Disease dataset (ROAD) [43]. The example data of the two datasets are shown in Figure 4. Each dataset was divided into two parts: a train-val part and a test part using the train-test-split function (TTSF) from the scikit-learn library. The details of the split are shown in Table 1. The KACD dataset contains 6400 2D MRI images from 6400 cases, and each case is assigned into one of four categories: Non-Demented, Very Mild Demented, Mildly Demented, and Moderately Demented. The ROAD contains 532 3D MRI images from 532 cases, and each case is assigned into one of three categories: Non-Demented, Mildly Demented, and Alzheimer’s disease. As shown in Table 1, the data set was separated into two parts, including a training-val set (TVS) for training and selection of model weights and an independent test set (TS) to evaluate the performance of the models. The TVS of the KACD contains 5121 2D MRI images, and the TS of the KACD contains 1279 2D MRI images. The TVS of the ROAD contains 300 3D MRI images, and the TS of the ROAD contains 232 3D MRI images.

### 4.2. Evaluation Metrics

The Kappa score (*Kappa*), sensitivity (*Sen*), specificity (*Spe*), precision (*Pr*), accuracy (*Acc*) and *F*1-score metrics were used to evaluate the performance of the proposed ADGNET comprehensively. The equations of the six metrics are as follows:

(7) pe=((TN+FN)×(TN+FP)+(TP+FP)×(TP+FN))/(N×N)

(8) p0=(TP+TN)/N

Given the definitions of *p*e and *p*0, the *Kappa* score is defined as follows:

(9) Kappa=(p0−pe)/(1−pe)

The sensitivity is defined as:

(10) Sen=TP/(TP+FN)

The specificity is defined as:

(11) Spe=TN/(TN+FP)

The precision is defined as:

(12) Pr=(TP)/(TP+FP)

The accuracy is defined as:

(13) ACC=(TP+TN)/(TP+TN+FP+FN)

The *F*1-score is defined as:

(14) F1−Score=2×Pr×Sen/(Pr+Sen)

where *TP* represents the true positive, *TN* represents the true negative, *FP* represents the false positive, and *FN* represents the false negative. Six evaluation metrics (*Kappa*, *Sen*, *Spe*, *Pr*, *Acc* and *F*1-score) were employed to evaluate the performance of the proposed ADGNET and other SOTA WSL-based methods. The *Kappa* is a statistical indicator of the stability of the model prediction. The *Sen* is related to the positive prediction rate and is a significant indicator in medical diagnosis. The *Spe* indicates the correctness of the model’s prediction and also has great significance in medical diagnosis. The *Pr* refers to the ability of the model to provide a positive prediction. The *Acc* is an indicator of the correctness of the model’s prediction. The *F*1-score is the harmonic mean of the *Pr* and *Sen*.

### 4.3. Experimental Results

The performance of the proposed ADGNET was evaluated using multimodal datasets (2D MRI images and 3D MRI images) to assess the generalization ability and transferability of the model with six metrics (*Kappa*, *Sen*, *Spe*, *Pr*, *Acc* and *F*1-score). Two sets of experiments were conducted (experiments A and B) to evaluate the performance of the proposed ADGNET. A bootstrapping method was used to calculate the empirical distributions of the boxplots. All experiments were conducted on the KACD dataset and ROAD dataset. An ablation study was also conducted to better demonstrate the effectiveness of the proposed framework. As shown in Table 2 and Table 3, the ADGNET (proposed) means the proposed framework as demonstrated in Figure 1; the ADGNET (no RSN) means the *Subnet2:RSN* as shown in Figure 1 was excluded while the rest of the proposed framework are retained and trained with the same amount of annotations; the ADGNET (no AM) means the *Attention Mechanism* as shown in Figure 2 was excluded while the rest of the proposed framework are retained and trained with the same amount of annotations. The training and inference processes were performed on four Nvidia GTX 2080Ti GPUs and Intel Xeon E5-2600 v4 3.60 GHz CPU using the Pytorch framework.

#### 4.3.1. Experiment A: Performance on the KACD Dataset (Comparison between the Proposed ADGNET, ResNeXt WSL and SimCLR)

**Figure 5:**
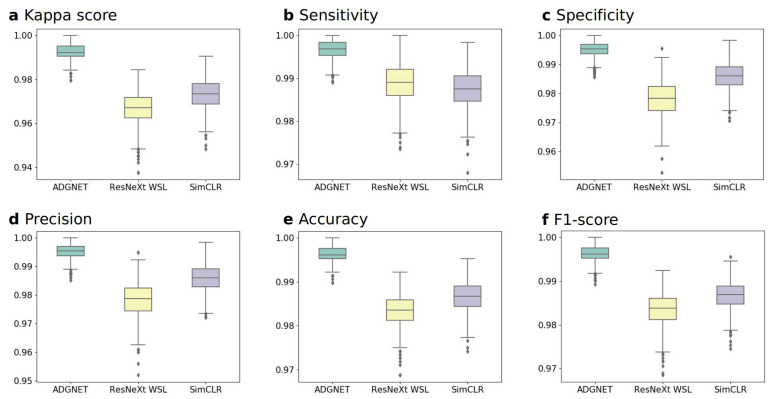
Boxplots of the six evaluation metrics of the models in experiment A. (**a**) kappa score. (**b**) sensitivity. (**c**) specificity. (**d**) precision. (**e**) accuracy. (**f**) F1-score.

In this experiment, we used the 2D MRI images from the KACD dataset to evaluate the models’ performances. The overall results of the six metrics for the proposed ADGNET, the ResNeXt WSL, and the SimCLR are listed in Table 2. The optimum performance was obtained by ADGNET, with an *F*1-score of 99.61%, followed by SimCLR (98.67%) and ResNeXt WSL (98.37%). The Acc was highest for ADGNET (99.61%), followed by SimCLR (98.60%) and ResNeXt WSL (98.36%). The *Pr*, *Spe*, *Sen* and *Kappa* of ADGNET were 99.53%, 99.53%, 99.69% and 99.22%, respectively. The values of the indices were higher than those of ResNeXt WSL (97.84%, 97.81%, 98.91% and 96.72%) and SimCLR (98.60%, 98.59%, 98.75% and 97.34%). The corresponding boxplots of the six evaluation metrics (*Kappa*, *Sen*, *Spe*, *Pr*, *Acc* and *F*1-score) of the models’ performance on the KACD dataset are shown in Figure 5.

#### 4.3.2. Experiment B: Performance on the ROAD Dataset (Comparison between the Proposed ADGNET, ResNeXt WSL and SimCLR)

**Figure 6:**
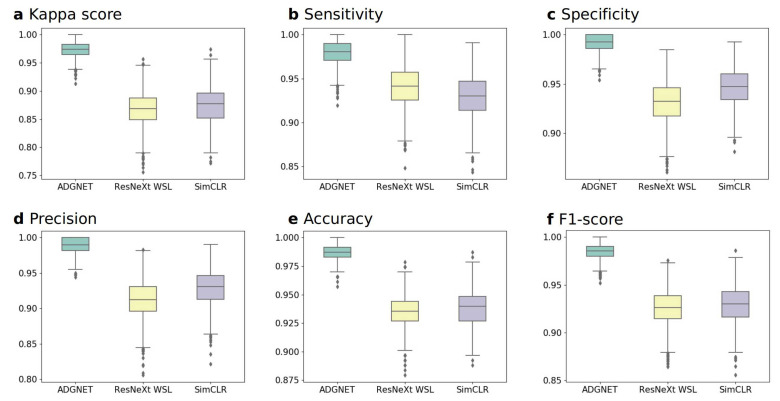
Boxplots of the six evaluation metrics of the models in experiment B. (**a**) kappa score. (**b**) sensitivity. (**c**) specificity. (**d**) precision. (**e**) accuracy. (**f**) F1-score.

In this experiment, we used the 3D MRI images from the ROAD dataset to evaluate the models’ performance. The overall result of the six metrics for the proposed ADGNET, the ResNeXt WSL, and the SimCLR are listed in Table 3. The best performance was obtained by the proposed ADGNET, with an *F*1-score of 98.49%, followed by SimCLR (93.00%) and ResNeXt WSL (92.61%). The Acc was highest for ADGNET (98.71%), followed by ResNeXt WSL (93.53%) and SimCLR (93.00%). The *Pr*, *Spe*, *Sen* and *Kappa* of ADGNET were 98.99%, 99.24%, 98.00% and 97.36%, respectively. The values of the indices were higher than those of ResNeXt WSL (91.26%, 93.18%, 94.00% and 86.87%) and SimCLR (93.00%, 94.70%, 93.00% and 87.70%). The corresponding boxplots of the six evaluation metrics (*Kappa*, *Sen*, *Spe*, *Pr*, *Acc* and *F*1-score) of the models’ performance on the ROAD dataset are shown in Figure 6.

#### 4.3.3. Training Details

The TVS of each dataset was split into 5 parts using a stratified sampling method. The model was trained using 20% of the labels. A 5-fold cross-validation was adopted to evaluate the performance of the trained model. The samples in the TS of each dataset were used to verify the performance of the proposed ADGNET.

## 5. Discussion

**Figure 7:**
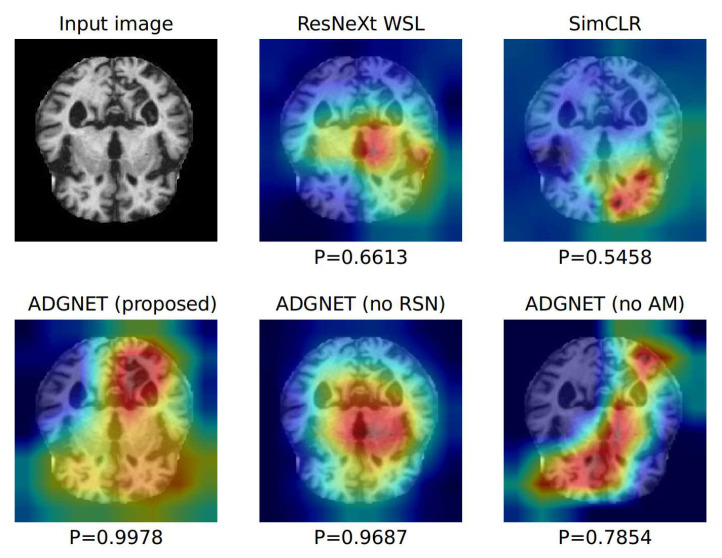
Visualization of heatmaps. We compare the visualization heatmaps of the ADGNET (proposed, no reconstruction sub-network (RSN) and no attention module (AM)), the ResNeXt WSL (weakly supervised learning), and the SimCLR. The heatmap visualization is calculated for the last convolutional outputs and P denotes the prediction score of each network for the ground-truth category.

We developed a CAD method for the identification and classification of AD in multi-modal brain imaging data (2D MRI and 3D MRI) using WSL-based DL techniques. Excellent performance was obtained by the proposed ADGNET based on six evaluation metrics, and the method proved superior to two SOTA WSL-based methods. The proposed ADGNET is a modular framework consisting of a backbone and the task subnets. We used a residual block in the design of the backbone network to retain the features while preventing degradation of the framework. We incorporated an AM into the backbone network to ensure the high discriminatory ability of the backbone network with a low computational cost. Unlike the conventional methods like Resnext WSL which assign the same weight to each channel, the proposed AM learned the channel weights of the input feature maps from the supervised information and the images for an improved feature representation of the samples. This helps the framework to focus more on the most discriminative part from the feature space. The feature vector obtained from the pooling map of the backbone network was flattened and sent to the two sub-networks. The CSN extracted the feature information directly from the vector and was optimized using the supervised information. The RSN encoded the vector to a new feature space using two FC layers and used a decoder to reconstruct the input images. The two FC layers and the backbone network comprised the encoder that was used for feature coding. The proposed decoder network consisted of the transposed convolution layer and the convolution layer. The objective of the transposed convolution layer was to learn the feature information for image reconstruction, and the convolution layer was used to adjust the number of channels and generate the final output. Unlike previous WSL-based methods (e.g., ResNeXt WSL and SimCLR), which have to be pre-trained on a large independent dataset and fine-tuned on the target dataset, the proposed ADGNET is trained in an end-to-end manner. The training process of the ADGNET is controlled by adjusting the weighting parameters. When $\lambda_{1}$ is zero, the network only learns the features from the images. When $\lambda_{2}$ is zero, the network only learns the features from the annotations. In this way, the large-scale unlabeled data can be fully utilized to help the proposed framework obtain more stable and representative features. Excellent performance was achieved by ADGNET based on the six statistical metrics for the multi-modal brain imaging datasets (KACD (2D MRI) and ROAD (3D MRI)). In order to intuitively analyze the experimental results, the heatmaps of the proposed ADGNET and the two SOTA models were demonstrated in Figure 7. As can be seen from Figure 7, the proposed ADGNET is able to capture key features while retaining more features by means of the AM and the RSN. While the ResNeXt WSL and SimCLR only use very limited features for prediction and their prediction scores are relatively low. Notably, the ADGNET’s prediction score is quite higher than ResNeXt WSL and SimCLR, which indicated that the ADGNET’s prediction is more reliable. The ADGNET has a promising potential as an auxiliary tool to assist in the diagnosis of AD due to its high performance, good stability, and cross-modal flexibility. Besides, medical diagnosis in a real situation is much more complex than in experimental environments, and sufficient and high-quality annotations are difficult to obtain. Therefore, the development of our proposed WSL-based DL methods is crucial for diagnosing conditions such as AD. However, the proposed ADGNET may also encounter some problems when the distribution of the data has extremely category imbalance. It is also important to develop effective generative frameworks to generate a large amount of effective data for compensation with WSL-based methods.

## 6. Conclusions

This study presented a unique WSL-based DL framework for the identification and classification of AD using multi-modal brain imaging data (2D MRI and 3D MRI). The proposed ADGNET provided excellent performances on six metrics (*Kappa*, *Sen*, *Spe*, *Pr*, *Acc* and *F*1-score), outperforming the two SOTA WSL-based models on two public datasets (KACD (2D MRI) and ROAD (3D MRI)) using limited annotation (only 20% of the labels). Most notably, the Kappa of the ADGNET was 0.9922 on the KACD dataset and 0.9736 on the ROAD dataset. These values were 2.50% and 1.88% higher than those of the two SOTA methods on the KACD dataset and 10.49% and 9.66% higher on the ROAD dataset, respectively.

The excellent performance achieved by ADGNET indicates that the proposed AM and the framework are suitable for the task and that the model is superior to the two SOTA WSL-based methods. The proposed AM module enabled the ADGNET to automatically assign different weights for different channels in the feature maps for a better capture of discriminative features. It is well-known that obtaining a large sample size and high-quality annotations of medical images is time-consuming and expensive. The introduction of sub-network for image reconstruction task help the ADGNET acquire effective features mining from large scale unlabeled data. Therefore, the development of WSL-based DL methods might represent a potential research direction to achieve accurate mining of massive medical data. In the future, the potential of this framework will be explored in-depth for other challenging tasks, including the detection of brain tumors and other major diseases.

## Figures and Tables

**Table 1:** Distribution of the Kaggle Alzheimer’s classification dataset (KACD) and Recognition of Alzheimer’s Disease dataset (ROAD).

KACD	Train-Val	Test	Total	ROAD	Train-Val	Test	Total
NoneDemented	2560	640	3200	NoneDemented	68	52	120
Very Mild Demented	1792	448	2240	Very Mild Demented	-	-	-
Mild Demented	717	179	896	Mild Demented	151	116	267
Moderate Demented	52	12	64	Alzheimer’s disease	81	64	145
Total	5121	1279	6400	Total	300	232	532

**Table 2:** Performance indices of the proposed ADGNET framework of the experiment A and the average performance of the two state-of-the-art (SOTA) models on the KACD dataset.

	KACD Dataset				
	ADGNET (Proposed)	ADGNET (No RSN)	ADGNET (No AM)	ResNeXt WSL	SimCLR
*Kappa* (95%CI)	0.9922	0.9781	0.9812	0.9672	0.9734
	(0.9844, 0.9984)	(0.9656, 0.9890)	(0.9703, 0.9906)	(0.9514, 0.9797)	(0.9609, 0.9844)
*Sen* (95% CI)	0.9969	0.9906	0.9922	0.9891	0.9875
	(0.9921, 1.0000)	(0.9824, 0.9970)	(0.9845, 0.9984)	(0.9804, 0.9955)	(0.9785, 0.9953)
*Spe* (95% CI)	0.9953	0.9875	0.9890	0.9781	0.9859
	(0.9890, 1.0000)	(0.9783, 0.9953)	(0.9799, 0.9955)	(0.9663, 0.9888)	(0.9769, 0.9937)
*Pr* (95% CI)	0.9953	0.9875	0.9891	0.9784	0.9860
	(0.9894, 1.0000)	(0.9780, 0.9953)	(0.9798, 0.9956)	(0.9670, 0.9889)	(0.9805, 0.9922)
*Acc* (95% CI)	0.9961	0.9891	0.9906	0.9836	0.9860
	(0.9922, 0.9992)	(0.9828, 0.9945)	(0.9851, 0.9953)	(0.9757, 0.9898)	(0.9805, 0.9922)
*F*1-score (95% CI)	0.9961	0.9891	0.9906	0.9837	0.9867
	(0.9922, 0.9992)	(0.9828, 0.9945)	(0.9849, 0.9956)	(0.9756, 0.9901)	(0.9806, 0.9922)

**Table 3:** Performance indices of the proposed ADGNET framework of the experiment B and the average performance of the two SOTA models on the ROAD dataset.

	ROAD Dataset				
	ADGNET (Proposed)	ADGNET (No RSN)	ADGNET (No AM)	ResNeXt WSL	SimCLR
*Kappa* (95% CI)	0.9736	0.9210	0.9473	0.8687	0.8770
	(0.9387, 1.0000)	(0.8696, 0.9654)	(0.9032, 0.9825)	(0.7986, 0.9300)	(0.8143, 0.9308)
*Sen* (95% CI)	0.9800	0.9600	0.9700	0.9400	0.9300
	(0.9500, 1.0000)	(0.9175, 0.9906)	(0.9310, 1.0000)	(0.8889, 0.9804)	(0.8764, 0.9770)
*Spe* (95% CI)	0.9924	0.9621	0.9773	0.9318	0.9470
	(0.9754, 1.0000)	(0.9274, 0.9924)	(0.9503, 1.0000)	(0.8824, 0.9699)	(0.9091, 0.9835)
*Pr* (95% CI)	0.9899	0.9505	0.9700	0.9126	0.9300
	(0.9674, 1.0000)	(0.9053, 0.9897)	(0.9347, 1.0000)	(0.8509, 0.9619)	(0.8775, 0.9780)
*Acc* (95% CI)	0.9871	0.9612	0.9741	0.9353	0.9300
	(0.9698, 1.0000)	(0.9353, 0.9828)	(0.9526, 0.9914)	(0.9009, 0.9655)	(0.9095, 0.9655)
*F*1-score (95% CI)	0.9849	0.9552	0.9700	0.9261	0.9300
	(0.9655, 1.0000)	(0.9246, 0.9817)	(0.9436, 0.9903)	(0.8832, 0.9608)	(0.8912, 0.9630)

## Data Availability

No new data were created or analyzed in this study. Data sharing is not applicable to this article.

## Abbreviations

The following abbreviations are used in this manuscript:

AD	Alzheimer’s Disease
ADGNET	Alzheimer’s Disease Grade Network
WSL	Weakly Supervised Learning
DL	Deep Learning
WHO	World Health Organization
MCI	Mild Cognitive Impairment
CT	Computed Tomography
PET	Positron Emission Tomography
MRI	Magnetic Resonance Imaging
CAD	Computer-Aided Diagnosis
CNN	Convolution Neural Network
SOTA	State-of-the-Art
AM	Attention Module
MTL	Multi-Task Learning
SIMO	Single-Input-Multi-Output
CSN	Classification Sub-Network
RSN	Reconstruction Sub-Network
GAP	Global Average Pooling
FC	Fully Connected
CAF	Channel Attention Factors
EWMO	Element-Wise Multiplication Operation
BN	Batch Norm
KACD	Kaggle Alzheimer’s Classification Dataset
ROAD	Recognition of Alzheimer’s Disease Dataset
TTSF	Train-Test-Split Function
TVS	Training-Val Set
TS	Testing Set
Sen	Sensitivity
Spe	Specificity
Pr	Precision
Acc	Accuracy
